# Learning loss and psychosocial issues among Thai students amidst the COVID-19 pandemic: the perspectives of teachers in the online classroom

**DOI:** 10.1186/s40359-023-01269-1

**Published:** 2023-08-16

**Authors:** Ittipaat Suwathanpornkul, Chawapon Sarnkhaowkhom, Manaathar Tulmethakaan, Panida Sakuntanak, Orn-uma Charoensuk

**Affiliations:** 1 Department of Educational Measurement and Research, Faculty of Education, Srinakharinwirot University, Bangkok, Thailand; 2 Department of Health Education and Behavioral Sciences, Faculty of Public Health, Mahidol University, Bangkok, Thailand

**Keywords:** COVID-19, Learning loss, Online classroom, Pandemic, Psychology, Psychosocial, Qualitative research, Student

## Abstract

**Background:**

The COVID-19 pandemic has had a broad influence on health and society across the globe, especially on the education sector as its educators have had to continue the provision of teaching and learning opportunities to their students online in the midst of pandemic. This has led to learning loss and psychological health issues among students, which is now being recognized worldwide. This study aims to explore the perceptions, perspectives, and experiences of teachers with regards to learning loss and psychological health issues among students during the COVID-19 pandemic in Thailand.

**Methods:**

Descriptive qualitative research was employed to gain a richer understanding of this interesting phenomenon. The experiences of twelve primary and secondary teachers were looked into by dividing them into several focus groups and interviewing them through video conferencing. All interviews were recorded, transcribed and analyzed through the use of content analysis.

**Results:**

The findings that include the perceptions, perspectives, and experiences among teachers focused on three main themes, which are teaching and Learning during the COVID-19 pandemic, learning loss caused by the COVID-19 pandemic, and support to deal with the psychosocial issues during the COVID-19 pandemic. These are then divided into fifteen sub themes.

**Conclusions:**

Through these findings, this study is able to provide an understanding of the perceptions, perspectives, and experiences of teachers regarding learning loss and psychosocial issues among students during the COVID-19 pandemic in Thailand. It also highlights the psychological issues that were met, the social and family support offered, ways how learning loss among students could have been prevented in the midst of the pandemic, and finally suggests what the education administrators and healthcare or public health sector administrators can do to enhance the quality of education and resilience skills of the students during and after the pandemic.

## Introduction

The COVID-19 pandemic was official reported to have commenced in Wuhan, the capital of Hubei Province, in the People’s Republic of China at the end of December 2019 [1]. After that, the pandemic spread to several areas across the globe. Thailand was officially announced as the first country with confirmed cases of the coronavirus 2019 disease outside of Mainland China on January 13, 2020 [1, 2]. After this in March 26, 2020, the Thai government declared a state of emergency to control the spread of COVID-19 [3, 4]. During the state of emergency, the education in Thailand became disrupted and all teaching and learning was transferred from face-to-face learning to online and distance learning [5]. Some of the crucial issues that resulted from this were learning loss, the worsening of the psychosocial health status of both the teachers and students, and an increase in the workload of teachers and students while teaching or studying [6, 7]. This occurred to primary and secondary school students across all education levels, cultures, socio-economic statuses, family backgrounds, and parts of the country since they were found to suffer from these consequences [5, 8–11].

The COVID-19 pandemic has had a broad influence on society, people’s quality of life, and the economic stability of many countries across the globe. Moreover, the COVID-19 pandemic caused educational institutions of all levels to discontinue classes for some periods of time. Consequently, the students did not learn wholly as they were supposed to through their new educational system [5, 9]. Teachers had to adjust their teaching techniques, learning management, and their teaching platform into a form of online learning or virtual classroom to maintain the continuation of their students’ learning [12, 13]. Apart from school administrators and teachers having to make substantial efforts to allocate resources to meet their students’ study needs, parents, guardians and related family members have also been required to support the learning of the students as well [14].

Due to the impact of the COVID-19 pandemic, the U.S. Department of Education, Office for Civil Rights [15] made interesting observations about the effectiveness of students learning online after collecting data from primary, secondary, and post-secondary students from the middle of March 2020 until mid-April 2021 when many U.S. schools conducted online teaching and learning. To sum up the research, the COVID-19 pandemic was found to negatively influence academic growth which can be seen through the learning outcomes for certain mathematics and reading courses not being met. Some grades showed a significant decline compared to the pre-epidemic situation. Moreover, it was reasoned that this was mainly due to the increasing inequality and opportunities regarding access to learning channels. For instance, some students faced technological issues and there were other technological difficulties that led to disruptions within the virtual classrooms. In addition, students were more exposed to the risk of suffering mental health, well-being, and psychosocial problems due to the lack of opportunities to acquire assistance when required [5, 6]. Also, students with learning disabilities who required specific academic support faced disruption due to the pandemic situation, resulting in more inequality [15, 16].

Learning loss was mainly caused by students having no commitment to online learning activities as well as limitations in learning management owing to issues with devices and the internet. Even though parents and students wished to return to the traditional classroom, some were still afraid of infection [17]. They mainly wished them to return because students spent less time on learning. Moreover, students were bound to their homes, leading to stress and anxiety which negatively affected the ability of these students to perform academic activities as they were less motivated to complete their learning activities [18, 19]. Furthermore, some were not ready to study online due to a lack of access to technology and an unsupportive home environment. Moreover, elementary and junior high school students were not yet ready to proceed with online learning due to the lack of technological equipment. Likewise, as mentioned previously, online learning was not suitable for students with special needs or those with disabilities, causing them to have behavioral and psychological problems when trying to focus on their lessons. Lastly, parents and students wanted to go back to the onsite class room as it would result in lower costs of living, as studying onsite had led to a decline in economic growth and employment [18].

The majority of learning losses experienced by students were seen in the areas of language skill acquisition and proficiency, including speaking, writing, reading fluency, and reading comprehension. Students also experienced problems with mathematics, such as when dealing with numbers, mathematical operations, solving mathematical problems, recognizing shapes, etc. [20–29]. Moreover, the chances of learning for some groups of students were reduced, and the number of students who dropped out in the middle of the semester saw an increase as well [15, 30]. In addition, a gap in knowledge between students with good and bad behaviors became very distinct [31]. Learning loss among students was therefore argued to be related to the students’ physical skills, social skills, emotional skills, mental health and well-being [20, 24]. Online knowledge acquisition therefore depended on the type of students and the issues they were facing.

The COVID-19 pandemic in Thailand consequently resulted in a prominent shift in the educational system through a shift in the learning management arrangement. For the academic year B.E. 2564 (2021), the Office of the Basic Education Commission of Thailand or OBEC [32] determined five types of learning management arrangement based on the suitability and context of each school, which were namely On-air, Online, On-demand, On-hand, and On-site. This meant schools that were closed were not limited to using online learning only. However, an assessment of the COVID-19 pandemic impact on education found that online learning was not as effective as traditional classroom learning, resulting in learning loss for students. This may have been due to the unpreparedness of the parents, lack of computer equipment, not all students having internet access, online learning requiring too much screentime, and the lacking of any interaction with friends causing stress for either the students or teachers [33–35]. Moreover, especially in families with a low household income, learning loss was especially prevalent, which in turn caused students to drop out of school [36, 37]. To handle this learning loss due to school closures as a result of COVID-19, more effective distance education may be required to help solve this problem [26, 38–40].

Apart from learning loss, the situation of the COVID-19 pandemic, which was a worldwide crisis, has led to teachers and students facing more pressure and stress from teaching and learning respectively [6, 7, 20, 24]. Moreover, for students and families who have been studying amidst the pandemic, the stress and difficulty of adapting to either the learning method or the pandemic has been even greater [41, 42]. Therefore, the COVID-19 pandemic has made it significant to conduct research among teachers who taught elementary, junior, and high school students amidst the pandemic to gain a deeper understanding of the perspectives, perceptions, and experiences of teachers when dealing with learning loss and the psychosocial issues of the students they encountered amidst the pandemic phenomenon, especially in Thailand so that the learning management arrangement can be adapted more quickly in the future and staff members will be more prepared to deal with policy changes, and the shortage of equipment and technology to prevent the learning and psychosocial issues and challenges occurring again among students and teachers. In particular, this greater comprehension is hoping to be gained through the use of an application of the social cognitive theory (SCT) of Bandura [43] as a tentative theoretical framework. This theory is based on the social learning theory and uses a qualitative descriptive approach to focus on the reciprocal interaction between the social contexts of the personal, environmental, and behavioral factors and the learning which people undergo. This qualitative descriptive approach focuses on gaining a greater understanding of the topic and a wide array of data or information from the key informants’ responses to open-ended and probing questions regarding the direct experiences or stories that have occurred to the informants to gain a greater understanding of the teachers’ perceptions, perspectives, and experiences when dealing with learning loss and the psychosocial issues of students they faced during the COVID-19 pandemic [44].

Furthermore, by looking at the findings of the research, the researchers will also be able to offer recommendations for the development of either the education, behavioral sciences, or public health fields. In particular, the ideas focused on the quality of teaching and learning development in the midst of the COVID-19 pandemic, may be applied to maintain the continuation of learning and prevent learning loss among students during this crisis and any future emergency situations. Moreover, any measures put forward to improve the students’ health, especially the mental health of students, may be adapted as well. For these reasons, this research aims to explore the perceptions, perspectives and experiences of teachers with regards to learning loss and the psychosocial issues of students encountered during the pandemic of COVID-19 in Thailand. This will be done by answering the following question: What are the perceptions, perspectives and experiences of teachers with regards to learning loss and the psychosocial issues of students faced by instructors during the COVID-19 pandemic in Thailand?

## Methods

### Design

This study employed a qualitative descriptive approach to gather information on the perceptions, perspectives, and experiences with regards to learning loss and the psychosocial issues of students faced amidst the COVID-19 pandemic among Thai primary and secondary teachers. The responses came from open-ended and probing questions [44]. The method of data collection was performed by using focus group interviews which involved Thai primary and secondary teachers that were held through the ZOOM video conferencing interview platform which at the time was in line with the COVID-19 pandemic prevention measures. The data was analyzed simultaneously by applying a content analysis method. This study followed the reporting guideline-Standards for Reporting Qualitative Research (SRQR) [45].

### Participants

The participant selection for this study was based on purposive sampling. The inclusion criteria when selecting the participants included the following: (a) The teachers had to come from primary, secondary or mixed primary and secondary schools; (b) Teachers had to be from schools affiliated with the Office of the Basic Education Commission (OBEC), the Provincial Administrative Organization (PAO) or the Bangkok Metropolitan Administration (BMA); (c) Teachers had to have performed teaching during the COVID-19 pandemic; (d) Teachers required experience of dealing with or managing learning loss or learning problems which students might have faced during the COVID-19 pandemic; (d) They should live permanently in Thailand and speak Thai; and (e) They should be willing to participate in this study. The exclusion criteria were being very sick or too ill to participate in this study. The 12 primary and secondary teachers who met the criteria were twice interviewed through one focus group. The participants were made up of seven female teachers and four male teachers. The work experience length among teachers was 3–23 years with the minimum length of service being 3 years and the maximum being 23 years. The participants’ schools covered each region of Thailand, such as the northern, eastern, western, northeastern, southern, central, and Bangkok metropolitan areas. Their schools’ affiliations were the Office of the Basic Education Commission (OBEC), the Provincial Administrative Organization (PAO), and the Bangkok Metropolitan Administration (BMA) respectively. The characteristics of the participants taking part in this study are summarized in Table 1.

**Table 1:** The characteristics of the participants (N = 12)

Participant number	Gender	Experiences (Years)	School	School size	Affiliation	Province	Region
Teacher 1	Female	5	Primary	Small	OBEC	Lamphun	Northern
Teacher 2	Female	9	Primary	Small	PAO	Samut Songkhram	Central
Teacher 3	Male	10	Primary	Medium	OBEC	Phetchaburi	Western
Teacher 4	Male	4	Primary	Medium	OBEC	Pattani	Southern
Teacher 5	Female	21	Primary	Large	PAO	Udon Thani	Northeastern
Teacher 6	Male	8	Primary	Large	BMA	Bangkok	Bangkok Metropolitan
Teacher 7	Female	6	Secondary	Large	OBEC	Nan	Northern
Teacher 8	Male	10	Secondary	Large	PAO	Sisaket	Northeastern
Teacher 9	Female	23	Secondary	Large	OBEC	Ang Thong	Central
Teacher 10	Male	10	Secondary	Large	PAO	Phatthalung	Southern
Teacher 11	Female	21	Secondary	Large	OBEC	Bangkok	Bangkok Metropolitan
Teacher 12	Female	3	Secondary	Large	OBEC	Pathum Thani	Bangkok Metropolitan

### Data collection

The data collection in this study was conducted to gain information and understanding of the perceptions, perspectives, and experiences regarding learning loss and the psychosocial issues of students faced in the midst of COVID-19 pandemic among Thai primary and secondary teachers through focus group interviews by using ZOOM meetings, a widely-used video conferencing platform. The focus group interview is one of the qualitative research methods which has been more widely used to help research participants exchange, share, and learn more about a certain topic [46]. In this study, the focus group interview was used to gain a deeper information and understanding of the phenomenon in question which allowed the Thai primary and secondary teachers, who the research participants were, to receive, learn, share, and exchange information together regarding the perceptions, perspectives, and experiences of learning loss and the psychosocial issues that the students faced during the COVID-19 pandemic. The benefit of focus groups is that when the participants talk about their own stories, they are more likely to express their own perceptions, perspectives, and experiences on the addressed topic. This can lead to a deeper understanding of the perceptions, perspectives, and experiences of learning loss and psychosocial issues of the participants. Observations with regards to the mood, tone, and expression of the interview participants were also made during the focus group interviews by the notetakers. At the beginning of each focus group interview, the principal investigator (PI) informed the participants about the study purpose and method, and also asked permission from the participants to confirm whether the data could be collected and recorded in either oral or written form. The data collection was ethically performed after approval of the ethical consideration in February 2022 by the research team, who had a vast array of experience of conducting focus group interviews and qualitative research. The focus group interviews were initiated with general open-ended questions and were followed up with specific open-ended or leading questions. The six main opened-ended questions asked in the focus group interviews were:

- “Would you mind telling us a little bit about yourself, and where are you working?”
- “Could you please tell me how your school managed the teaching and learning during the COVID-19 pandemic? (planning, designing, and learning management; measurement and evaluation; materials, tools and learning resources etc.)”.
- “When did you start facing problems when working or teaching your students during the COVID-19 pandemic?”
- “Could you please describe your situation with learning loss during the pandemic through your perceptions and experiences?”
- “Could you please tell me what you believe the causes or related factors are for learning loss among your students during the COVID-19 pandemic?”
- “Please, inform me more about your experiences of dealing with the students’ learning loss or teaching and working problems during the outbreak. What were the outcomes?”

The focus group interviews took around 120 to 150 min each time and the data were transcribed verbatim within 1–2 days after the focus group interviews. Throughout this whole process, the data and information of the informants was kept in anonymity by the research team.

### Data analysis

The collected data for this study was analyzed simultaneously after the focus group interviews, while the verbatim transcription was performed within 1–2 days after the focus group interviews. The collected data was analyzed based on the inductive content analysis (ICA) approach suggested by Vear and Gillam [47], which is seen as a meaningful and well-suited analytical approach to be applied to health-related research. Moreover, this approach is known to be used for small scale research, and research topics that had not been looked into yet, as none of the previous studies had dealt with the phenomenon regarding the perceptions, perspectives, and experiences of Thai teachers with regards to learning loss and the psychosocial issues encountered during the COVID-19 pandemic [47, 48]. The data analytical process followed the five steps suggested by Vear and Gillam [47] to analyze the perceptions, perspectives, and experiences of Thai teachers with regards to the learning loss among students and the psychosocial issues faced during the COVID-19 pandemic. These steps were: (a) reading and familiarizing oneself with the text or data (b) decoding the data in the first round and identifying the bigger picture (c) decoding the data in the second round and developing subcategories and fine-grained codes (d) refining of subcategories and fine-graining of the decoding (e) synthesizing and interpretating the data [47]. Afterwards, once the data analysis was completed, the research team would scrutinize the rigour or trustworthiness of the transcripts and analyze them.

### Rigour and trustworthiness

The rigour and trustworthiness of the findings of this study were established to reassure others that the findings could be trusted by utilizing the Guba and Lincoln approach [49] that followed the subsequent aspect: (a) credibility; the verbatim transcription was completed within a day to two days after the focus group interviews after which the preliminary themes were sent to all participants by the researchers to reach agreement on the themes and for the participants to check the accuracy of the themes. Moreover, the triangulation approach was undertaken by the research team, and a multi method for the data collection process was used to assure the data sources and methodology used were reliable. (b) dependability; the researchers applied various tools and instruments to collect the data. Ways to collect the data included data forms to collect information on the demographics of participants, focus group interview guidelines, a voice recorder, and note taking forms. The research team also collaborated to ensure that entire data was either collected or recorded, (c) confirmability; the data collection and analysis was systematically performed by the research team and the data was checked, investigated, and compared as part of demonstrating an audit trail throughout this study, and finally (d) transferability; the researchers were dedicated to providing detailed and reliable information on this insightful phenomena and also provide coverage of this phenomena at the same time. Therefore, the research team carefully provided an audit trail and carefully completed the data analysis by analyzing entire the data to gain a thoroughgoing understanding of the perceptions, perspectives, and experiences of teachers with regards to the learning loss among students and the psychosocial issues of students encountered during the COVID-19 pandemic in Thailand.

## Results

The perceptions, perspectives, and experiences of primary and secondary teachers regarding learning loss and the psychosocial issues of students faced during the COVID-19 pandemic in Thailand fell into 3 main major themes and 15 sub-themes as shown in Table 2.

**Table 2:** The main themes and sub-themes of the study

Main themes	Sub-themes
1. Teaching and learning during the COVID-19 pandemic	1.1 Usage of various teaching and learning approaches
	1.2 Variety of tools, materials and technologies used for the teaching and learning
	1.3 Reforming of school policies, learning management arrangements, and evaluation of the learning outcomes
2. Learning loss during the COVID-19 pandemic	2.1 Loss of cognitive skills and knowledge linkages
	2.2 Loss of positive attitudes toward learning and school subjects
	2.3 Loss of desirable characteristics of students
	2.4 Loss of literacy and numeracy skills
	2.5 Loss of scientific and laboratory skills
	2.6 Loss of life skills, relationships, and collaboration skills
3. Psychosocial issues and support provided during the COVID-19 pandemic	3.1 Changes in the motivation, attention, readiness, and confidence of students
	3.2 Emotions, relationships, and mental health of students
	3.3 Decrease of compassion and support from parents
	3.4 Teacher’s adjustment and teacher-student relationships
	3.5 Educational materials and technological support
	3.6 Policy, measures, and management of the COVID-19 pandemic in schools

### Teaching and learning during the COVID-19 pandemic

The transition in teaching and learning in Thai schools in the midst of pandemic occurred in three ways: some schools applied various teaching and learning approaches to enhance the seamlessness and quality of teaching; some schools implemented a variety of tools, materials and technologies to aid with the teaching and learning; whereas some schools reformed the policies, learning management arrangements, and evaluation of the learning outcomes.

### *Usage of various teaching and learning approaches*

The COVID-19 pandemic has caused all educational institutions in Thailand to adjust their learning management arrangements based on the national measures to prevent the spread of COVID-19. Teachers at primary and secondary levels provided information on how they shifted from face-to-face learning to a variety of other learning management arrangements including teaching through TV (On-air) via satellite; through the internet (Online) via various channels, such as ZOOM Meetings and the Line Application; On-demand teaching via DLTV; On-hand teaching for students who are not equipped with technological equipment through the use of books, worksheets, and exercises for students which they take home and submit to their teachers at school; and on-site teaching by choosing students from even or odd numbers to study in school on certain days to provide students with continuous learning opportunities.

*“The learning management arrangements while learning online have been different compared to the regular learning arrangements. The program was a little flexible but included all courses. There was only one Social Studies course and the focus was on the main subjects, namely Science, Math, and the Thai language, which were arranged in the morning. The schedule was made up of 3 periods per day, but the main courses were in the morning. The school had several learning management arrangements, such as on-air, online, on-demand, on-hand, and on-site. It was up to the teacher which one to choose although fully on-site was not available (laughing)…”* (Elementary school teacher I).

### *Variety of tools, materials and technologies used for the teaching and learning*

Teachers provided information on their use of tools and technology while teaching. Most of them employed technological tools to conduct online learning. These tools were both free of charge and offered through various platforms including ZOOM Meeting, Google Classroom, Google Meet, YouTube, Facebook, and the Line Application. They helped deliver comprehensive online learning. Moreover, the teachers also offered exercises, books, and worksheets as media for on-hand teaching.

*“…in the early phase of online learning, the school helped to provide training to teachers to enable them to use ZOOM and Google Meetings as the main online learning programs… We had experience of it being conducted 100% online in 2020. It seemed convenient as we were following the Government guidelines to offer online learning. However, various applications were added as some teachers started using YouTube, Live, and other applications to manage their courses to be more interesting and fun.”* (Secondary school teacher IV).

### *Reforming of school policies, learning management arrangements, and evaluation of the learning outcomes*

Teachers at both primary and secondary levels debated shifts in policies, administration, and evaluation of their own educational outcomes to respond to the shifts in learning management arrangements in various forms. They said that schools had been willing to alter their policies and that the administration of the educational institutes had become more flexible and reliant on the available tools, the various perspectives on assessment methods and the students’ academic performances. Some teachers mentioned that their school had offered support for more internet signal installations and green backgrounds for teachers to conduct their online teaching while some teachers said that schools required teachers to complete the course material within a certain period so that students would not have to spend too much time learning online. In addition, some institutes had a policy to not check the names of students for attendance while some schools decided to reduce the students’ workload by 50%.

*“…This semester, we will conduct a 100% online class. We will reduce the periods. Before we had 9 periods, but we will divide them in half. Period 1–4 will be week A while period 5–9 will be week B. Then, we will switch A to B like this. The learning time might be reduced but students do not have to sit and suffer in front of the computer for a long time…”* (Secondary school teacher VI).

### Learning loss during the COVID-19 pandemic

Learning loss caused by the COVID-19 pandemic among primary and secondary students occurred across six dimensions: loss of cognitive skills and knowledge linkages; loss of positive attitudes toward learning and school subjects; loss of desirable characteristics among students; loss of literacy and numeracy skills; loss of scientific and laboratory skills; and loss of life skills, relationships, and collaboration skills.

### *Loss of cognitive skills and knowledge linkages*

The recent learning management arrangements during the COVID-19 pandemic resulted in learning loss among elementary school students as it became clear that the comprehension of the lessons taught by the teachers had decreased. The decreased ability of some students to recognize and understand lessons was observed in their questions and answers, their behavior, and the quality of their completed worksheets or assignments when compared to traditional learning. In addition, most secondary school teachers witnessed cognitive problems among their students and established a link between the declining level of student knowledge and the use of various learning management arrangements as a substitute for traditional learning. Students had the ability to learn new things but at a slower rate and they were unable to link the knowledge that had been taught in a previous period to the new lesson and apply it. Therefore, there was a clear decrease in the amount of knowledge that was maintained and understood compared to when teaching in normal situations.

*“…Students gained new academic and theoretical knowledge at a slower rate, and they lacked the ability to make connections with previous knowledge that had been taught. Moreover, sometimes we already knew that they had learned something, but they could not apply the previous knowledge to the new knowledge or they lacked the persistence to study by themselves and remember it. For example, this was taught last week and if we did not review it, they would forget since they did not show the persistence to remember the content covered…”* (Secondary school teacher II).

### *Loss of positive attitudes toward learning and school subjects*

Most students had a negative attitude toward the changing of their learning and course arrangements and expressed this through behaviors, such as absenteeism and their inattention to study, leading to many students dropping out of the education system. Some teachers recounted the resonance of students who thought online learning was not their world. For instance, sitting in front of a computer screen triggered a question among several students about what they were doing and when they had any doubt about what the teacher had taught, they did not dare to ask since they were afraid that they would waste their classmates’ time. Furthermore, they did not want to face their discomfort of revealing their home environment while learning online.

*“…With regards to the change in students’ attitudes, they all said they didn’t want to study online since they felt it wasn’t their world. They wanted to interact with their peers, play and relax. They were uncomfortable sitting and watching something even if it was to gain new knowledge. When they had questions, they did not dare to ask since they were afraid of wasting their peers’ time. Sometimes, they did not dare to ask the teacher, and the teacher could not walk around to observe the students’ facial expressions. For instance, they could not see the puzzled faces and most the time the whole room was silent when learning online. If we were in the traditional classroom, we would know if they didn’t understand the lessons, so we could explain it again, but when studying online, only some students turned on the camera as they were allowed to choose whether to turn on their camera or not, so we could not see everyone’s face.”* (Secondary school teacher V).

### *Loss of desirable characteristics of students*

Teachers also provided information on the changing in the students’ characteristics as online learning was believed to have had an impact on students. Especially among elementary school students, teachers had noticed that the students were less reluctant to answer the questions by themselves when learning online. Students tended to look at their parents’ faces first since they were sat with their parents and did not want to answer the question in front of them, which was different from teaching in the traditional classroom. Some teachers noticed that students started copying their peers’ answers too when they had to complete a worksheet or assignment by taking photos or sharing information in a LINE group before sending it to the teacher. Some teachers found that their students’ enthusiasm for learning had decreased compared to the normal situation before COVID-19. Students who once had to wake up early to go to school were in a ready-to-learn environment that may not be conducive to learning and they were easily distracted from learning. Some teachers gave the insight that the students’ responsibility and discipline had worsened. Once they submitted work on time, but now they submitted their work late and lacked responsibility for the assigned tasks.

*“…When learning online, the students stay with their parents. Students with issues would look at their parents first, and not answer when asked a question. Before the pandemic they were close to me in the classroom and could talk to me, but when I asked questions in an online class, all students were silent, they did not answer, and turned to look at their parents who would then answer all for them.”* (Elementary school teacher I).

### *Loss of literacy and numeracy skills*

Most teachers mentioned the problem that the level of the students’ skills had declined during the COVID-19 pandemic, especially with regards to reading and mathematics skills which were considered to be very important skills for elementary school students as they were the basic skills to continue learning in other courses and at the next level. Most students saw a drop in their reading skills and there was not enough time to repeat the reading lessons for students due to time limitations. Turning to mathematics, some were unable to solve math problems and they could not consult their parents to find the answers as their parents did not understand the subject matter, resulting in more stress and unwillingness to study that subject. Although some teachers said that learning management during COVID-19 led to the growth of students’ IT skills, it was clear that the skills related to reading and mathematics had been reduced too much compared to learning in normal situations.

*“…The Thai language, mathematics and reading skills for students are seen as crucial basic skills for elementary school students to advance in other courses. As I said, after evaluating what they sent us, we were not sure if it was truly their work or not. It’s not like when we’re in the real classroom, right?”* (Elementary school teacher II).

### *Loss of scientific and laboratory skills*

Most teachers mentioned a decline in and a lack of science skills among their students. Under normal situations, students were taught scientific skills through laboratory practice. During the COVID-19 pandemic, students were taught through video or by looking at the teacher’s experiment while some teachers said that they could not teach students cooking skills, such as baking, home economics, or computer programming during the COVID-19 pandemic. A teacher from the Thai Dance course described the decreased chance of improving the dance skills of students as they were missing practice. It was normally practiced through teaching and face-to-face guidance from a teacher under normal circumstances.

*“…I teach chemistry, and first of all, they did not do any lab practice at all. They would lack the skills to use scientific tools. Grade 10-11-12, students would be unable to gain practice in titration or electrochemistry, and they would completely lack practice when trying to use scientific tools.”* (Secondary school teacher III).

### *Loss of life skills, relationships, and collaboration skills*

Most teachers provided information that more students were facing issues developing life skills, relationships with other students, and working with others compared to the learning situation in a normal environment. Teachers noticed that some students were not satisfied with their studies. To illustrate, they recorded video clips and sent them to their teachers in tears, thus causing pity for them in the hope that the teachers would communicate with their parents to aid them more. Some teachers said that the relationships among students and between students and teachers had worsened which could be observed by looking at the changes in the students’ utterances. For example, they started to use curse words as they found it easier to express dissatisfaction with their peers. It was also found that students rarely interacted and worked with others since while staying at home, they rarely met people or some people only stayed in their room or dormitory by themselves, thus diminishing the students’ life skills as well.

*“…When observing the classroom, students were more stubborn when teaching them, but we could still teach them. Now, it turns out that they changed their way of words when talking to others. They started talking to friends with profanity. When dissatisfied, they would show it instantly. This is a behavioral change as they were left with technology without advice. That’s it. Most core skills in the past were gained by working together and showing more adaptability, but now they had to gain those skills by themselves.”* (Elementary school teacher V).

### Psychosocial issues and support provided during the COVID-19 pandemic

Both primary and secondary school teachers faced challenges, problems, and issues while doing their work in the midst of the COVID-19 pandemic, especially with regards to adapting to the psychological changes of their students, dealing with student issues and offering the students the correct support.

### *Changes in the motivation, attention, readiness, and confidence of students*

Most teachers noticed behavioral expressions of burnout from students who were not interested in studying or completing assigned activities as they showed a decreased motivation to study. Some teachers mentioned a lack of self-confidence or insecurity when asking or answering the students’ questions. Teachers noticed that the students were afraid to ask questions and chose to ask teachers in private because they were afraid that peers would joke with them about their stupidity. Some students did not dare to do activities or exercises assigned by the teacher until their classmates had completed them and shared the answers with the other students. This led to a shift in the learning behavior of the students which differed from studying in a class at school under normal circumstances which in turn affected the understanding of the lesson and the learning ability of the students. Some students had a reduced or lack of motivation to study while some were experiencing fatigue from studying. On the other hand, the students’ unreadiness to study when living in a home environment that was not conducive to learning caused an unwanted feeling to study. Furthermore, some students at home needed to help their parents with chores or work and earn a living, so they might have needed to miss classes or would have been unable to attend the entire lesion as when studying in a traditional classroom at a school. This had a negative impact on the comprehension of the students and led to a decrease in the learning continuity as well as learning opportunities.

*“…The reason for the lack of motivation and self- direction came from themselves. There was no motivation since they didn’t ask for people’s help. No one motivated them. Some parents sometimes blame their children when they cannot complete the assigned tasks, and the parents will seek the advice of the teacher. As teachers, we have to call through Line and tell them not to put blame on the children but gradually give them time and encouragement to complete the assigned tasks.”* (Elementary school teacher III).

### *Emotions, relationships, and mental health of students*

In the recent learning management arrangement during the COVID-19 pandemic, most teachers noticed and faced negative emotions from their students. Most students underwent boredom, unhappiness, lack of motivation, and burnout during lessons during certain situations. This was different to when some teachers provided information in the normal situation as then students tended to engage in activities with their peers and teachers. Some students who were in the school’s folk song band spent most of their time with teachers and peers in the band together under normal circumstances, causing them to have interactions with friends and teachers, which gave them the opportunity to engage in activities and build mutual relationships between peers and teachers. Some teachers mentioned the mental health of students had worsened as some experienced stress and pressure from their workload, their failure to adapt to change all the time as well as the increased burden and responsibilities from being at home. Moreover, the COVID-19 Pandemic prevented students from seeking counseling with teachers as they previous did in normal situations.

*“…I think that the intimacy between the teacher and the student has completely gone. In the past, students could come to me for consulting and we became closer, and they would tell us everything right away. As this kind of phenomenon has now gone, we don’t even know how they are. They must be stressed. The opportunity that they come to consult us or to see us is now very rare.”* (Secondary school teacher III).

#### *Decrease of compassion and support from parents*

Parents’ empathy, understanding, and support for learning during the COVID-19 pandemic were variously based on each family’s background. Most parents had a relatively low level of understanding of the changes of the dynamic learning management arrangement as a substitute for the traditional classroom. As a result, students were not interested in learning as the majority of parents were not interested in checking their attendance, offering support, participating in activities, or checking if the assigned tasks from the teachers were done due to having insufficient time cause of work duties. However, some parents were strict with their children at home and offered support to the students to engage them in their school activities. Maybe, the assigned tasks from teachers were too much, causing students to not understand the lesson, activities, or exercises assigned by the teacher. There was also pressure and tension occurring in the family when studying at home. For some parents, when they saw their children sit at home online, they often asked them to help with chores or work in the family business during the students’ study time. Some parents thought that while learning online at home, students often played games or did not pay attention to studying, so they gave them tasks while some parents were less likely to prioritize their students’ studying and thought helping the family was more important. On the other hand, some parents were strict with their children’s studying, resulting in stress because of pressure.

*“…Based on the parents’ perspectives, they may not understand their children’s learning methods. Based on online home visits and parent meetings, it became clear that some parents did not understand their children’s learning method. Also, there was a profound lack of support. Even if they had all the resources, they did not support the children as they should. For those who lacked resources, they lacked learning equipment… Some parents lost their jobs, so they had no income at all. They therefore asked their children for help, such as looking at their younger siblings while the parents could find more sources of income. Some students even had to go out to work. They said, “I have to go help my father with work today.”* (Secondary school teacher II).

### *Teacher’s adjustment and teacher-student relationships*

Teachers said that some teachers had a strong ability to adapt to the new teaching methods. This was because they could utilize technological media for their learning management arrangement to gain the students’ attention. On the other hand, some had a strict teaching style by assigning work to students for which they had to study and prepare reports. Other teachers had students take notes or write down what the teacher taught during the class and they had to hand it to the teacher. As a result of the teacher’s methods of adapting to the learn management situation, some secondary school students underwent stress and showed a lack of interest, leading to learning loss. Moreover, the relationship between teachers and students has headed into a negative direction. Online learning has provided teachers and students with distance and teachers have not been able to guide their students as they had done in the past as a result of that. This includes a greater lack of understanding with regards to the lessons taught by teachers. Teachers used to observe behaviors and facial expressions to notice if a student was unable to follow a lesson and then gave suggestions to students in a timely manner. Instead, while studying online, there was more distance between the student and the instructor and the students had to spend more time on discovering and solving their problems, thus causing them to miss out on learning opportunities, especially in terms of students not gaining the required characteristics to be successful students, them not understanding the lessons and the students not getting the various skills required.

*“…The new generation of teachers was able to use technology while some teachers only had limited capabilities to use technology due to their age and experience. As a result. they may not be too comfortable teaching online, so it becomes all tense, and not flexible as they want their teaching to be strictly perfect. As a result, if the class is not perfect, this would lead to students having a bad attitude towards their courses as well. Moreover, children will also think that online learning is bad because of one course which in turn could affect other courses.”* (Secondary school teacher VI).

#### *Educational materials and technological support*

Family support varied as not all families possessed the technological devices that could facilitate online learning for their children as the facilitation of technological devices was dependent on different socioeconomic backgrounds and the parents’ occupations. The technological devices provided by some of the parents were so limited at a level where the students only had a parent’s mobile phone to study online. If parents had to use their mobile phone to communicate, students had to leave class for a while to allow their parents to use the mobile phone. Some teachers also mentioned that some students had a poor internet signal while studying online. Some students lived in highland areas or mountains, so they had limited internet reception. Due to the unavailability of learning materials, technology, and internet signals, students lacked continuity in their learning while some went missing during their studies, which led to learning loss among these students again due to the COVID-19 pandemic.

*“…Students live in remote areas and they have to attend class online. If they do not have an internet connection, they have to go to the village hall. In some families with many siblings, when studying, students have to use the phone to study together. Parents didn’t have the money to support the online studying part. Some schools, however, solved the problem by having the teachers survey their students to determine if the students were ready to learn online. A report was written up for every class to determine which families were having problems providing technological devices for their children. At some schools, there was a group of children who didn’t have phones at all, and the school solved the problem by buying phones for these children for the purpose of studying.”* (Secondary school teacher III).

#### *Policy, measures, and management of the COVID-19 pandemic in schools*

A shift in school policies and administration during the COVID-19 pandemic was rapid and able to be made at any moment, causing students to feel confused and unable to plan their learning and life. They also could not focus on their learning as they should. Some teachers mentioned that some school policies and administrations lacked flexibility, including policies on teacher assessment, school quality assurance, and scheduling. For instance, some schools reduced the duration of the study day to allow students to focus more on the main courses, causing some subjects to not be taught. Therefore, students could not learn other subjects they might have had more interest in as in normal circumstance.

*“…As for the school, I’m actually quite suspicious of the school’s policy as it keeps changing and the policy can be controvertible as one policy states that we should conduct tests every week. Sometimes, this affects students. They might be confused about whether they have to go to school and what the situation will be like next week. They cannot plan their life. This also provoked problems with the school administration.”* (Secondary school teacher V).

## Discussion

Based on the research findings when narrating the perceptions, perspectives, and experiences with regards to learning loss among students and the psychosocial issues of students witnessed in the midst of COVID-19 pandemic among Thai primary and secondary teachers, the three main major themes and fifteen sub-themes generated novel interesting research findings that deviated from previous research as they focused on the learning loss of students during the school summer break while some other results may have just presented the learning loss in a general context or before the COVID-19 pandemic. This research demonstrated the perceptions, perspectives, and experiences among Thai primary and secondary teachers with regards to a particular context, which focused on the recent situation of the COVID-19 pandemic, in which teachers who were working in education were required to provide teaching and learning to students under the several conditions and challenges offered by the pandemic across the globe [12, 50].

Regarding the situation of learning management arrangements during the COVID-19 pandemic, educational institutions provided various types of learning management arrangements with diverse tools and technologies [12, 13, 51]. The school policies, administration, and evaluation of learning strategies had been modified to respond to the negative educational impacts of the pandemic effectively [52, 53]. The findings in this section were in accordance with the guidelines provided for the modifying of the learning management arrangements offered by the Office of the Basic Education Commission (OBEC), Thailand [32]. For the academic year B.E. 2564 (2021), the Office of the Basic Education Commission of Thailand (OBEC) determined the types of learning management arrangements into five methods based on the suitability and specific context of each school, which were the learning management arrangements throughout the on-air learning through digital and network televisions; online learning; on-demand learning; on-hand teaching and learning; and on-site learning so that schools that were closed were not limited to using the online learning method solely [32].

Learning loss was clearly witnessed through the decline in learning achievements of primary and secondary students during the COVID-19 pandemic. These findings were based on qualitative data, through which teachers expressed that their students had experienced a loss in learning opportunities, which was shown through a decline in their knowledge, knowledge linkage, and scientific and practical skills. The findings were consistent with the Asian Development Bank [20]; Azim Premji Foundation [21]; Blaskó et al. [22]; Cardinal [23]; Fitzpatrick et al. [24]; Kaffenberger [25]; Locke et al. [26]; Raymond [27]; Salciccioli [28]; and Zierer [29] noting that the majority of learning losses experienced by students were found to be in one’s language skills and proficiency, which included speaking, writing, reading fluency, and reading comprehension. Learning loss was also found with mathematics, since there were issues with identifying numbers, conducting mathematical operations, solving mathematical problems, and recognizing shapes, etc. [54]. It was also seen that students from different levels faced different levels of learning loss. However, this learning loss for these subjects can be further explained in the context of the Thai education administration that Foreign Languages, Science, and Mathematics are courses for which students have to rely on their knowledge, understanding, and practice as well as the teacher’s ability to transfer knowledge and skills to their students [21, 26]. Therefore, students were found to have a greater learning loss for these types of courses than for other courses which could be taught by parents or learned by students themselves.

A change in the students’ characteristics was found as students seemed more reluctant to answer questions, and showed a lack of participation when learning, while there was also a decline in their life skills, mental health, emotional states, relationships, and ability to work with others as well as a decline in positive attitudes towards learning and courses. The findings were consistent with DiPietro et al. [18] who reported that students spent less time on learning. The students were bound to their homes, leading to stress and anxiety which negatively affected the ability of students to complete assignments and perform academic activities [24, 55–57]. This led to the Asian Development Bank [20] and Fitzpatrick et al. [24] stating that learning loss was related to the physical skills, social skills, emotional skills, mental health, and well-being of the students. Moreover, some of the changes in characteristics and learning loss of students were related to unprepared learning management arrangements, and a lack of equipment and technology for learning [22, 58, 59]. This was consistent with Cho et al. [17] who found distance learning was often limited in terms of equipment and the internet.

For the limitations of this study, there were a few limitations regarding this research that should be declared and considered when performing data interpretation. First, due to the COVID-19 pandemic, the participants were recruited through video and telephone calls which could have led to limitations. However, the research team attempted to perform and record the focus group interviews through video conferencing interviews to avoid the validity of the data interpretation being affected as the research team could not observe the expressions, moods, emotions, and non- verbal actions of the participants during the focus group interviews when performing telephone interviews. Second, only a small number of teachers who worked in small and medium size schools were included in this study, which may have had an influence on the richness and coverage of the information and the results as not all the point of views and experiences of the primary and secondary teachers had been taken into account. However, this research nonetheless provides fascinating findings that help gain an insightful understanding of the perceptions, perspectives, and experiences of teachers with regards to learning loss among students and the psychosocial issues of students faced amidst the COVID-19 pandemic in Thailand, which may contribute to the improvement of the child, the family and their psychological health, the educational policies, school management, learning loss prevention, and the quality of teaching and learning.

## Conclusions

The perceptions, perspectives, and experiences of teachers with regards to learning loss among students and the psychosocial issues of students encountered during the COVID-19 pandemic in Thailand provide some interesting and insightful findings that people can learn from. The findings revealed the current teaching and learning transformation responses to the COVID-19 pandemic in Thailand for which various teaching and learning approaches have been applied, a variety of materials and technologies have been implemented, and the school policies, the learning management arrangements, and evaluation of the learning outcomes have been adapted. Additionally, the perceived learning loss among the students caused by the pandemic according to the teachers was generally seen through the loss of the students’ cognitive skills and knowledge linkages, positive attitudes toward learning and school subjects, desirable characteristics of students, literacy, numeracy, scientific and laboratory skills, and life skills, relationships, and collaborations. These issues should be considered and managed during any educational policy formation and teachers should be prepared to ensure that the students become familiar with the new learning arrangement so that they can continue learning while not missing out. Furthermore, the findings also showed the students psychosocial issues from the teacher’s perspective as they highlighted changes in motivation, attention, emotions, relationships, the mental health of students. There was also a decrease in compassion and support from parents, teachers-student relationships, and technological support offered by schools. Therefore, it is believed that the students should be supported with their education and mental health by not only the educational sector, but also the healthcare sector. It is also believed that the strategies for prevention of learning loss and promotion of psychological well-being of the students and teachers should be used to enhance the students’ health and strengthen the quality of education. For further research, the researchers should focus on applying these findings as empirical evidence and a basis to design and develop a model or intervention to recover the students’ learning losses and enhance the mental health among students and teachers in the post-pandemic era according to their needs, perceptions, perspectives, experiences, and contexts.

## Data Availability

The datasets generated and/or analyzed during the current study are not publicly available due to ethical restrictions but are available from the corresponding author on reasonable request.
